# Supplementary material for: Cost-effectiveness analysis of a multiplex lateral flow rapid diagnostic test for acute non-malarial febrile illness in rural Cambodia and Bangladesh
Source: Lancet Reg Health Southeast Asia. 2024 Mar 16;23:100389. doi: 10.1016/j.lansea.2024.100389 (PMC10958476; doi:10.1016/j.lansea.2024.100389)
Supplement: Supporting Information S1 [file mmc1.pdf]

Supporting Information S1

RECOMMENDED INTERPRETATION AND MANAGEMENT FOR POSITIVE TESTS

Positive multiplex LF-RDT component	Recommended interpretation	Recommended management
Dengue	Patient has dengue	Referral to hospital for admission
Enteric fever	Patient has enteric fever	Ciprofloxacin
CRP (>40 mg/L)	Patient has a bacterial infection (not enteric fever if enteric fever test is negative, otherwise enteric fever if enteric fever test also positive)	Amoxicillin unless enteric fever test also positive, in which case ciprofloxacin
None	Patient has a viral infection	No antibiotics

CAMBODIA

Model Settings

Reset all model inputs

Model settings

Time horizon	Lifetime	
DSA/PSA default variation	20.00%	
Number of PSA iterations	1,000	
Willingness-to-pay threshold<br>Note: Adjust if changing country	$357	*Woods et al. Inflated to 2022 values - Bangladesh: 51-723 (avg 388). Cambodia: 56-658 (avg 357). Original values - Bangladesh: 30-427 (avg 229). Cambodia: 44-518 (avg 281). 2013 data.*
Perspective	Societal	
Country	Cambodia	

Population characteristics

	Value	Source
Average age	28	SEACTN data (21/03/2022-21/03/2023 - Bangladesh: 28.34 ;Cambodia: 34.21)
Proportion of adults	79.05%	SEACTN data (21/03/2022-21/03/2023)
Proportion of children 5-15 years	17.14%	SEACTN data (21/03/2022-21/03/2023)
Proportion of children <5 years	3.81%	SEACTN data (21/03/2022-21/03/2023)

Scenario analysis inputs (in place of the above)

Fever of unknown source

Proportion of adults	82.84%	SEACTN data (21/03/2022-21/03/2023)
Proportion of children 5-15 years	13.64%	SEACTN data (21/03/2022-21/03/2023)
Proportion of children <5 years	3.52%	SEACTN data (21/03/2022-21/03/2023)

Introduction

Settings

Results

SA Settings

DSA Results

PSA Results

Decision Tree

Decision tree calculations

Intervention: Clinical assessment only

Branch	True disease	Diagnosis	Outcome	Probability	Diagnostic costs	Hospitalisation costs	Treatment costs	Societal costs	**Total costs**	YLD	YLL	**DALYs**
1	Enteric fever	Enteric fever	Survive	1.27%	$0.00	$0.23	$0.02	$0.06	**$0.31**	0.0094	*0.0000*	**0.0094**
2	Enteric fever	Enteric fever	Die	0.01%	$0.00	$0.00	$0.00	$0.00	**$0.00**	0.0001	0.0038	**0.0039**
3	Enteric fever	Other	Survive	1.26%	$0.00	$0.37	$0.03	$0.02	**$0.42**	0.0098	*0.0000*	**0.0098**
4	Enteric fever	Other	Die	0.01%	$0.00	$0.00	$0.00	$0.00	**$0.00**	0.0001	0.0051	**0.0052**
5	Dengue	Dengue	Survive - observed in hospital	11.56%	$0.00	$1.20	*$0.00*	*$0.00*	**$1.20**	*0.0000*	*0.0000*	**0.0000**
6	Dengue	Other	Survive - not observed in hospital	0.87%	$0.00	$0.00	*$0.00*	*$0.00*	**$0.00**	*0.0000*	*0.0000*	**0.0000**
7	Other viral	Enteric fever	Survive	17.28%	$0.00	$1.19	$0.29	$0.80	**$2.28**	*0.0000*	*0.0000*	**0.0000**
8	Other viral	Dengue	Survive	47.69%	$0.00	$4.94	*$0.00*	*$0.00*	**$4.94**	*0.0000*	*0.0000*	**0.0000**
9	Other viral	Other bacterial	Survive - antibiotic prescribed	1.79%	$0.00	$0.00	$0.06	$0.04	**$0.10**	*0.0000*	*0.0000*	**0.0000**
10	Other viral	Other viral	Survive - no antibiotic prescribed	2.36%	$0.00	$0.00	*$0.00*	*$0.00*	**$0.00**	*0.0000*	*0.0000*	**0.0000**
11	Other bacterial cause	Other bacterial	Survive - antibiotic prescribed	11.16%	$0.00	$0.00	$0.37	$0.28	**$0.64**	*0.0000*	*0.0000*	**0.0000**
12	Other bacterial cause	Other viral	Survive - no antibiotic prescribed	4.74%	$0.00	$0.00	*$0.00*	*$0.00*	**$0.00**	*0.0000*	*0.0000*	**0.0000**
				**100.00%**	**$0.00**	**$7.93**	**$0.76**	**$1.21**	**$9.90**	**0.0194**	**0.0089**	**0.0282**

Percentage receiving antibiotics 31.51%

Inappropriate antibiotics 20.02%

Intervention: Novel multiplex + clinical assessment

Branch	True disease	Diagnosis	Outcome	Probability	Diagnostic costs	Hospitalisation costs	Treatment costs	Societal costs	**Total costs**	YLD	YLL	**DALYs**
1	Enteric fever	Enteric fever	Survive	2.15%	$0.11	$0.39	$0.04	$0.10	**$0.63**	0.0160	*0.0000*	**0.0160**
2	Enteric fever	Enteric fever	Die	0.02%	$0.00	$0.00	$0.00	$0.00	**$0.00**	0.0001	0.0065	**0.0066**
3	Enteric fever	Other	Survive	0.38%	$0.02	$0.11	$0.01	$0.01	**$0.15**	0.0029	*0.0000*	**0.0029**
4	Enteric fever	Other	Die	0.00%	$0.00	$0.00	$0.00	$0.00	**$0.00**	0.0000	0.0015	**0.0016**
5	Dengue	Dengue	Survive - observed in hospital	10.56%	$0.53	$1.09	*$0.00*	*$0.00*	**$1.62**	*0.0000*	*0.0000*	**0.0000**
6	Dengue	Other	Survive - not observed in hospital	1.86%	$0.09	$0.00	*$0.00*	*$0.00*	**$0.09**	*0.0000*	*0.0000*	**0.0000**
7	Other viral	Enteric fever	Survive	3.46%	$0.17	$0.24	$0.06	$0.16	**$0.63**	*0.0000*	*0.0000*	**0.0000**
8	Other viral	Dengue	Survive	3.46%	$0.17	$0.36	*$0.00*	*$0.00*	**$0.53**	*0.0000*	*0.0000*	**0.0000**
9	Other viral	Other bacterial	Survive - antibiotic prescribed	9.95%	$0.50	$0.00	$0.33	$0.25	**$1.07**	*0.0000*	*0.0000*	**0.0000**
10	Other viral	Other viral	Survive - no antibiotic prescribed	52.25%	$2.61	$0.00	*$0.00*	*$0.00*	**$2.61**	*0.0000*	*0.0000*	**0.0000**
11	Other bacterial cause	Other bacterial	Survive - antibiotic prescribed	11.77%	$0.59	$0.00	$0.39	$0.29	**$1.27**	*0.0000*	*0.0000*	**0.0000**
12	Other bacterial cause	Other viral	Survive - no antibiotic prescribed	4.13%	$0.21	$0.00	*$0.00*	*$0.00*	**$0.21**	*0.0000*	*0.0000*	**0.0000**
				**100.00%**	**$5.00**	**$2.20**	**$0.81**	**$0.81**	**$8.82**	**0.0191**	**0.0080**	**0.0271**

Percentage receiving antibiotics 27.34%

Inappropriate antibiotics 13.69%

Model Results

Introduction

Settings

Results

SA Settings

DSA Results

PSA Results

Cost-Effectiveness Results

	Diagnostic costs	Hospitalisation costs	Treatment costs	Societal costs	**Total Costs**	YLD	YLL	**DALYs**	% of cases correctly diagnosed	% of patients prescribed antibiotics
Clinical assessment	$0.00	$7.93	$0.76	$1.21	**$9.90**	0.0194	0.0089	**0.0282**	32.88%	31.51%
Multiplex LF-RDT	$5.00	$2.20	$0.81	$0.81	**$8.82**	0.0191	0.0080	**0.0271**	90.84%	27.34%

Incremental Results

	Diagnostic costs	Hospitalisation costs	Treatment costs	Societal costs	**Total Costs**	YLD	YLL	**DALYs averted**	% of cases correctly diagnosed	% of patients prescribed antibiotics	**ICER ($/DALY averted)**
Multiplex LF-RDT vs. Clinical assessment	$5.00	-$5.74	$0.05	-$0.40	**-$1.08**	0.0003	0.0009	**0.0012**	57.96%	-4.16%	-$940.95

l

Scenario Analyses

Run Scenario

Introduction

Settings

Results

SA Settings

DSA Results

PSA Results

Sensitivity and Specificity Scenario Analyses

Dengue multiplex			
Sensitivity	Specificity	NMB	Threshold cost-effective price
0.9	0.6	$1.42	$6.42
0.8	0.7	$2.05	$7.05
0.7	0.8	$2.69	$7.69
0.6	0.9	$3.33	$8.33

Enteric fever multiplex			
Sensitivity	Specificity	NMB	Threshold cost-effective price
0.9	0.6	-$5.08	-$0.08
0.8	0.7	-$2.71	$2.29
0.7	0.8	-$0.34	$4.66
0.6	0.9	$2.03	$7.03

CRP multiplex			
Sensitivity	Specificity	NMB	Threshold cost-effective price
0.9	0.6	-$0.04	$4.96
0.8	0.7	$1.44	$6.44
0.7	0.8	$2.91	$7.91
0.6	0.9	$4.39	$9.39

All 3 tests			
Sensitivity	Specificity	NMB	Threshold cost-effective price
0.9	0.6	-$8.03	-$3.03
0.8	0.7	-$4.83	$0.17
0.7	0.8	-$1.09	$3.91
0.6	0.9	$3.17	$8.17

NMB	$1.49

BANGLADESH

Model Settings

Reset all model inputs

Introduction

Settings

Results

SA Settings

DSA Results

PSA Results

Model settings

Time horizon	Lifetime	
DSA/PSA default variation	20.00%	
Number of PSA iterations	1,000	
Willingness-to-pay threshold<br>Note: Adjust if changing country	$388	*Woods et al. Inflated to 2022 values - Bangladesh: 51-723 (avg 388). Cambodia: 56-658 (avg 357). Original values - Bangladesh: 30-427 (avg 229). Cambodia: 44-518 (avg 281). 2013 data.*
Perspective	Societal	
Country	Bangladesh	

Population characteristics

	Value	Source
Average age	28	SEACTN data (21/03/2022-21/03/2023 - Bangladesh: 28.34 ;Cambodia: 34.21)
Proportion of adults	79.05%	SEACTN data (21/03/2022-21/03/2023)
Proportion of children 5-15 years	17.14%	SEACTN data (21/03/2022-21/03/2023)
Proportion of children <5 years	3.81%	SEACTN data (21/03/2022-21/03/2023)

Scenario analysis inputs (in place of the above)

Fever of unknown source

Proportion of adults	82.84%	SEACTN data (21/03/2022-21/03/2023)
Proportion of children 5-15 years	13.64%	SEACTN data (21/03/2022-21/03/2023)
Proportion of children <5 years	3.52%	SEACTN data (21/03/2022-21/03/2023)

Decision Tree

Introduction
Settings
Results
SA Settings
DSA Results
PSA Results

Decision tree calculations

Intervention: Clinical assessment only

Branch	True disease	Diagnosis	Outcome	Probability	Diagnostic costs	Hospitalisation costs	Treatment costs	Societal costs	**Total costs**	YLD	YLL	**DALYs**
1	Enteric fever	Enteric fever	Survive	1.27%	$0.00	$0.23	$0.02	$0.06	**$0.31**	0.0094	*0.0000*	**0.0094**
2	Enteric fever	Enteric fever	Die	0.01%	$0.00	$0.00	$0.00	$0.00	**$0.00**	0.0001	0.0038	**0.0039**
3	Enteric fever	Other	Survive	1.26%	$0.00	$0.37	$0.03	$0.02	**$0.42**	0.0098	*0.0000*	**0.0098**
4	Enteric fever	Other	Die	0.01%	$0.00	$0.00	$0.00	$0.00	**$0.00**	0.0001	0.0051	**0.0052**
5	Dengue	Dengue	Survive - observed in hospital	11.56%	$0.00	$1.20	*$0.00*	*$0.00*	**$1.20**	*0.0000*	*0.0000*	**0.0000**
6	Dengue	Other	Survive - not observed in hospital	0.87%	$0.00	$0.00	*$0.00*	*$0.00*	**$0.00**	*0.0000*	*0.0000*	**0.0000**
7	Other viral	Enteric fever	Survive	17.28%	$0.00	$1.19	$0.29	$0.80	**$2.28**	*0.0000*	*0.0000*	**0.0000**
8	Other viral	Dengue	Survive	47.69%	$0.00	$4.94	*$0.00*	*$0.00*	**$4.94**	*0.0000*	*0.0000*	**0.0000**
9	Other viral	Other bacterial	Survive - antibiotic prescribed	1.79%	$0.00	$0.00	$0.06	$0.04	**$0.10**	*0.0000*	*0.0000*	**0.0000**
10	Other viral	Other viral	Survive - no antibiotic prescribed	2.36%	$0.00	$0.00	*$0.00*	*$0.00*	**$0.00**	*0.0000*	*0.0000*	**0.0000**
11	Other bacterial cause	Other bacterial	Survive - antibiotic prescribed	11.16%	$0.00	$0.00	$0.37	$0.28	**$0.64**	*0.0000*	*0.0000*	**0.0000**
12	Other bacterial cause	Other viral	Survive - no antibiotic prescribed	4.74%	$0.00	$0.00	*$0.00*	*$0.00*	**$0.00**	*0.0000*	*0.0000*	**0.0000**
				**100.00%**	**$0.00**	**$7.93**	**$0.76**	**$1.21**	**$9.90**	**0.0194**	**0.0089**	**0.0282**

Percentage receiving antibiotics	31.51%
Inappropriate antibiotics	20.02%

Intervention: Novel multiplex + clinical assessment

Branch	True disease	Diagnosis	Outcome	Probability	Diagnostic costs	Hospitalisation costs	Treatment costs	Societal costs	**Total costs**	YLD	YLL	**DALYs**
1	Enteric fever	Enteric fever	Survive	2.15%	$0.11	$0.39	$0.04	$0.10	**$0.63**	0.0160	*0.0000*	**0.0160**
2	Enteric fever	Enteric fever	Die	0.02%	$0.00	$0.00	$0.00	$0.00	**$0.00**	0.0001	0.0065	**0.0066**
3	Enteric fever	Other	Survive	0.38%	$0.02	$0.11	$0.01	$0.01	**$0.15**	0.0029	*0.0000*	**0.0029**
4	Enteric fever	Other	Die	0.00%	$0.00	$0.00	$0.00	$0.00	**$0.00**	0.0000	0.0015	**0.0016**
5	Dengue	Dengue	Survive - observed in hospital	10.56%	$0.53	$1.09	*$0.00*	*$0.00*	**$1.62**	*0.0000*	*0.0000*	**0.0000**
6	Dengue	Other	Survive - not observed in hospital	1.86%	$0.09	$0.00	*$0.00*	*$0.00*	**$0.09**	*0.0000*	*0.0000*	**0.0000**
7	Other viral	Enteric fever	Survive	3.46%	$0.17	$0.24	$0.06	$0.16	**$0.63**	*0.0000*	*0.0000*	**0.0000**
8	Other viral	Dengue	Survive	3.46%	$0.17	$0.36	*$0.00*	*$0.00*	**$0.53**	*0.0000*	*0.0000*	**0.0000**
9	Other viral	Other bacterial	Survive - antibiotic prescribed	9.95%	$0.50	$0.00	$0.33	$0.25	**$1.07**	*0.0000*	*0.0000*	**0.0000**
10	Other viral	Other viral	Survive - no antibiotic prescribed	52.25%	$2.61	$0.00	*$0.00*	*$0.00*	**$2.61**	*0.0000*	*0.0000*	**0.0000**
11	Other bacterial cause	Other bacterial	Survive - antibiotic prescribed	11.77%	$0.59	$0.00	$0.39	$0.29	**$1.27**	*0.0000*	*0.0000*	**0.0000**
12	Other bacterial cause	Other viral	Survive - no antibiotic prescribed	4.13%	$0.21	$0.00	*$0.00*	*$0.00*	**$0.21**	*0.0000*	*0.0000*	**0.0000**
				**100.00%**	**$5.00**	**$2.20**	**$0.81**	**$0.81**	**$8.82**	**0.0191**	**0.0080**	**0.0271**

Percentage receiving antibiotics	27.34%
Inappropriate antibiotics	13.69%

Model Results

Introduction

Settings

Results

SA Settings

DSA Results

PSA Results

Cost-Effectiveness Results

	Diagnostic costs	Hospitalisation costs	Treatment costs	Societal costs	**Total Costs**	YLD	YLL	**DALYs**	% of cases correctly diagnosed	% of patients prescribed antibiotics
Clinical assessment	$0.00	$7.93	$0.76	$1.21	**$9.90**	0.0194	0.0089	**0.0282**	32.88%	31.51%
Multiplex LF-RDT	$5.00	$2.20	$0.81	$0.81	**$8.82**	0.0191	0.0080	**0.0271**	90.84%	27.34%

Incremental Results

	Diagnostic costs	Hospitalisation costs	Treatment costs	Societal costs	**Total Costs**	YLD	YLL	**DALYs averted**	% of cases correctly diagnosed	% of patients prescribed antibiotics	**ICER ($/DALY averted)**
Multiplex LF-RDT vs. Clinical assessment	$5.00	-$5.74	$0.05	-$0.40	**-$1.08**	0.0003	0.0009	**0.0012**	57.96%	-4.16%	-$940.95

l

Scenario Analyses

Run Scenario

Introduction

Settings

Results

SA Settings

DSA Results

PSA Results

Sensitivity and Specificity Scenario Analyses

Dengue multiplex			
Sensitivity	Specificity	NMB	Threshold cost-effective price
0.9	0.6	$1.42	$6.42
0.8	0.7	$2.05	$7.05
0.7	0.8	$2.69	$7.69
0.6	0.9	$3.33	$8.33

NMB $1.49

Enteric fever multiplex			
Sensitivity	Specificity	NMB	Threshold cost-effective price
0.9	0.6	-$5.08	-$0.08
0.8	0.7	-$2.71	$2.29
0.7	0.8	-$0.34	$4.66
0.6	0.9	$2.03	$7.03

CRP multiplex			
Sensitivity	Specificity	NMB	Threshold cost-effective price
0.9	0.6	-$0.04	$4.96
0.8	0.7	$1.44	$6.44
0.7	0.8	$2.91	$7.91
0.6	0.9	$4.39	$9.39

All 3 tests			
Sensitivity	Specificity	NMB	Threshold cost-effective price
0.9	0.6	-$8.03	-$3.03
0.8	0.7	-$4.83	$0.17
0.7	0.8	-$1.09	$3.91
0.6	0.9	$3.17	$8.17
